# Outcomes and complications of fibular head resection

**DOI:** 10.1007/s11751-012-0133-8

**Published:** 2012-03-31

**Authors:** D. K. Agarwal, S. Saseendar, D. K. Patro, J. Menon

**Affiliations:** 1 Department of Orthopedics, Jawaharlal Institute of Postgraduate Medical Education and Research (JIPMER), Puducherry, India; 2 40, Manakula Vinayagar Street, Sri Kailasa Nagar (ECR), Lawspet, Puducherry, 8 India

**Keywords:** Fibula, Bone transplantation, Morbidity, Joint laxity, Fibular regeneration

## Abstract

The fibular head is often used as donor graft material for reconstruction of defects of the distal radius. However little is known on the safety of such a procedure. This report describes the long-term donor-site morbidity following the procedure. Fourteen patients who underwent simple or marginal resections of the proximal fibula between 1990 and 2007 were reviewed. Subjective donor-site morbidity, knee and ankle range of motion and instability, presence of sensory or motor function loss, gait and fibular regeneration were assessed. The mean age at surgery was 25 years; six were male, eight were female and the mean follow-up was 11 years. Abnormal clinical findings were present in 10 patients (71.4 %): nine patients (64.3 %) had Grade 2 varus laxity at the knee confirmed by stress radiographs; one had sensory loss in the distribution of the superficial peroneal nerve. Patients with varus laxity had significantly higher mean age at surgery than those without varus laxity (*p* = 0.001). None had deformity at the knee or ankle. The range of joint movements was normal. All had a normal tibiotalar angle and none had proximal migration of the fibula. One patient demonstrated near-complete regeneration of the fibula. Donor-site morbidity following simple and marginal resection of the proximal fibula is acceptable. Older patients had a higher risk of demonstrable varus laxity at the knee but proximal fibula resection in children appears to be safe.

## Introduction

The fibula is a common donor site for patients undergoing bone reconstruction. Its length, structure and predictable vascular pedicle make it an ideal cortical bone graft [1, 2]. The proximal fibula is often resected for use in reconstruction of the defects of distal radius after excision of tumours [3–5]. There are few studies which report the morbidity after proximal fibula resection for malignant bone tumours, and little is known of the safety of proximal fibula resection for the reconstruction of bone defects [6–9]. The present study aims to assess the safety of proximal fibula resection by assessing patients undergoing simple or marginal resection of the proximal fibula.

## Patients and methods

Hospital records of patients who underwent proximal fibular resection for various indications between 1990 and 2007 were accessed. Patients undergoing simple resection of the proximal fibula for bone transplantation or marginal resection for benign tumours of the proximal fibula were included in the study. Patients undergoing wide resection of the proximal fibula for malignant tumours or aggressive benign tumours were excluded. Nineteen patients satisfied the inclusion criteria and were called for review. Fourteen patients were available for follow-up and were included in the study.

Subjective donor-site morbidity was determined with a questionnaire which recorded presence of pain, swelling, stiffness, weakness, instability, numbness, limp, restriction in daily activities and cosmesis. Clinical examination included assessment of knee and ankle range of motion, varus, valgus, anteroposterior and rotatory instability at the knee, ankle instability, sensory or motor loss and gait.

### Radiological assessment

All patients had routine anteroposterior radiographs to assess the length of the resected fibula and of the distal remnant and a standing anteroposterior radiograph of both ankles. Those patients who had clinical signs of varus instability of the knee underwent varus stress radiographs of the knee. The stress radiographs of both knees were taken with varus stress applied at 15–20° of knee flexion and with the patient supine. The lateral knee joint space was measured in the varus stress films. A difference of more than 5 mm compared with the normal side was considered as significant [10].

The anteroposterior radiograph of the ankle was used to study proximal migration of the distal remnant of fibula and to assess for valgus deformity. Proximal migration was measured as the distance between the tip of the lateral malleolus and the distal tibial articular surface in comparison with the opposite side [11]. The tibiotalar angle was measured to assess ankle valgus [11, 12]. A change of 5° or more was regarded as a valgus deformity [12].

All patients had a normal contralateral limb. Gait was assessed clinically. The ability to walk on heels, walk on the outside foot and springing or hopping on donor leg was also assessed. Patients with findings of instability were asked to walk on a ramp with a side slope of 20° to assess the effect of instability on gait.

## Results

The mean age of patients at surgery was 25 years (range 8–59 years). There were six male and eight female patients. The right side was operated in seven patients and left side in another 7. The follow-up ranged from 3 to 20 years (mean 11 years 1 month).

Seven patients underwent a ‘simple’ resection for reconstruction of a bone defect following upper limb surgery, while another seven underwent a ‘marginal’ resection for a benign tumour of the proximal fibula (Table 1). ‘Simple’ resection consisted of a subperiosteal resection of the proximal part of the fibula after detachment of the fibular collateral ligament and the biceps femoris from their insertions. ‘Marginal’ excision entailed an en bloc resection of the benign tumour through its pseudocapsule [7]. The stump of the collateral ligament and the tendon of the biceps femoris were reattached to the adjacent soft tissues after both procedures. The length of fibula resected ranged from 8 to 19.5 cm (mean 11.5 cm). The mean percentage of fibula resected was 31.4 % (23–49 %). The length of the distal remnant ranged from 20 to 29 cm (mean 24.9 cm).

**Table 1:** Summary of findings following proximal fibula resection

Patient no.	Age at surgery/sex	Side	Diagnosis	Length of follow-up	Resected fibula		Length of distal remnant	Regeneration	Donor-site morbidity	
					Length (cm)	% of Total			Symptoms	Clinical findings
1	38/M	R	ABC (R) Proximal fibula	3 years	9	24	29	N	–	Varus laxity
2	15/M	R	ABC (R) Proximal fibula	5 years 7 months	11.5	30	27	N	–	–
3	23/F	R	GCT (R) Proximal fibula	6 years 1 months	8.5	27	27.5	N	–	Varus laxity
4	10/M	R	Chondroblastoma (R) fibula	7 years 7 months	8	23	26.5	N	–	–
5	35/F	R	GCT (R) proximal fibula	10 years 8 months	8.5	23	28	N	–	Varus laxity
6	10/M	R	Osteochondroma (R) fibula	11 years 1 months	8.0	23	26.5	N	–	–
7	08/F	L	ABC (L) proximal fibula	17 years 7 months	8.0	27	21.5	N	–	–
8	20/M	L	GCT (R) distal radius	18 years 7 months	14.5	38	23.5	N	Occasional pain at donor site	Varus laxity
9	59/M	L	Chondrosarcoma (R) humerus	7 years 5 months	19.5	49	20.0	N	–	Varus laxity
10	33/F	L	GCT (L) distal radius	13 years 2 months	12.5	35	23.5	N	–	Varus laxity
11	22/F	L	GCT (L) distal radius	14 years 7 months	11.5	32	24.5	N	–	Varus laxity
12	25/F	R	GCT (L) distal radius	10 years 4 months	11.5	32	25.0	N	Occasional pain and swelling	Varus laxity
13	36/F	L	GCT (L) distal radius	10 years 7 months	14.0	39	21.5	N	Numbness foot	Varus laxity Decreased sensation in superficial peroneal nerve
14	20/F	L	GCT (L) distal radius	20 years	15	38	24.5	Y	–	–

### Subjective donor site symptoms

At final follow-up, two patients reported mild occasional pain at the donor site. One patient complained of numbness in the distribution of the superficial peroneal nerve but none felt disabled by the symptoms and did not seek treatment for their complaints. There were no cosmetic problems from the scars. None of the patients had a limp, difficulty in walking or running or instability of the knee or ankle.

### Clinical findings

There were abnormal clinical findings on examination in 10 of the 14 patients (71.4 %): nine patients (64.3 %) had Grade 2 varus laxity at the knee and one patient had sensory loss in the distribution of the superficial peroneal nerve. The age at surgery for the patients with varus laxity ranged from 20 to 59 years (mean—32.3 ± 12.1), while for those without varus, it ranged from 8 to 20 years (mean—12.6 ± 4.9). The mean age at surgery was significantly higher in those with varus laxity (*p* = 0.001).

There was no significant difference in the duration of follow-up between patients with varus laxity (125.9 ± 56 months) and those without varus laxity (148.4 ± 75 months, *p* = 0.534) suggesting that the difference in follow-up duration did not influence the incidence of varus laxity. There was no deformity of the knee or ankle at inspection in the standing position; the range of movement of the knee and ankle was normal. None of the patients had ankle instability.

### Gait

All patients were able to walk on heels and on the outer border of the foot without any discomfort. All patients were able to walk comfortably on a side slope of 20° without any signs of instability.

### Radiological assessment

**Fig. 1:**
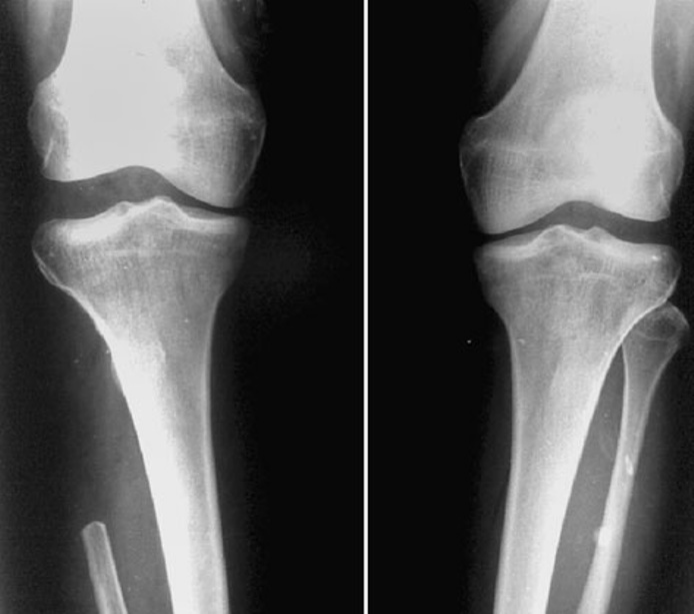
Varus stress radiograph of both knees showing a significant increase in lateral joint space after resection of the proximal fibula on the right side

Varus stress radiographs of patients with knee laxity demonstrated an increase in the lateral joint space of >5 mm as compared to the opposite side (Grade 2 instability, Fig. 1). None had a difference of more than 10 mm (Grade 3 instability).

No patient had significant difference in the tibiotalar angle as compared to the contralateral normal limb. None had proximal migration of the fibula.

**Fig. 2:**
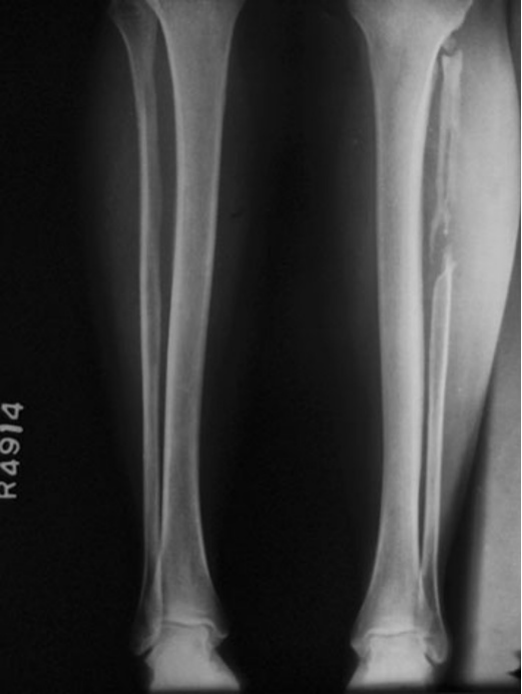
Proximal fibula regeneration after resection

It was noted that one patient who underwent proximal fibula transplantation to the distal radius at age 20 had near-complete regeneration of the fibula (Fig. 2). None of the other seven patients who underwent subperiosteal resection for bone transplantation demonstrated any evidence of such regeneration.

## Discussion

The fibula is an integral part of the ankle and knee joints and serves as an attachment for ligaments, the interosseous membrane and muscles of the lower extremity. Various biomechanical and cadaver studies have demonstrated the role of fibula in weight transmission and for normal function of the knee and ankle [13–17].

Babhulkar et al. reviewed 104 patients who underwent resection of the fibula for various reasons. Twenty-six of these patients had resection of the proximal fibula for reconstruction of an excised distal radius, and none had any demonstrable instability of the knee [20]. Pho et al. [21] also reported no instability after resection of the fibular head in three patients.

In contrast, abnormalities in the motion of knee, ankle and foot have been demonstrated after fibula removal in such biomechanical studies [17, 18]. In their cadaver study, Uchiyama et al. [19] found that the whole fibula, including the head of the fibula, was essential for the stability of the ankle joint complex.

Murray and Schlafly reported lateral ligament laxity in nine of 18 patients who underwent proximal fibular resection despite reattaching the tendons and ligaments to the remnant of the proximal fibula via drill holes [4]. Anderson and Green studied the functional deficit following fibulectomy for bone graft in 10 patients of whom two underwent resection of the proximal fibula. They had reattached the biceps femoris tendon and the fibular collateral ligament to the proximal tibia. One of the two patients had grade 1+ laxity on clinical examination and 5 mm opening on Genucom examination as compared to the opposite knee [6]. Draganich et al. reported the effects of resection of proximal fibula on the stability of the knee and on gait in their series of six patients. The fibular collateral ligament and the tendon of biceps femoris had been reattached to ligamentous and capsular structures. Based on instrumented analysis, they reported significantly increased anterior translation and varus and valgus rotation as compared to the contralateral limb. They suggested that biceps femoris imparted a posteriorly directed force to the tibia and the iliotibial band and that detaching the biceps femoris, a dynamic restraint to anterior displacement of the tibia, resulted in the demonstrable anterior tibial translation [7].

Bickels et al. analysed the outcomes of 24 patients who underwent proximal fibula resection for benign aggressive and malignant tumours. The lateral collateral ligament was reattached to the lateral tibial metaphysis using a staple with the knee in 20° flexion. Three patients (13 %) had grade 1 instability and one (4 %) had grade 2 instability. The authors recommend stapling of the lateral collateral ligament to the proximal tibial metaphysis as a safe and reliable technique to reestablish knee stability after resection of the proximal fibula [8].

Recently, Dieckmann et al. reported the outcomes of proximal fibula resection in 47 malignant and 10 benign tumours of the proximal fibula. Of 45 patients who required resection of the lateral ligament complex, 41 underwent reconstruction with an anchor or transosseous suture of the remaining biceps femoris tendon and ligament. Thirteen of the 45 patients (28.9 %) developed knee instability and required treatment with revision of the lateral ligament complex or through use of orthoses [9].

In this series, a large proportion (64.3 %) of the patients had clinically and radiologically demonstrable knee joint laxity but none had severe or symptomatic instability to warrant treatment. The higher incidence of demonstrable knee instability is likely to be due to attachment of the collateral ligament and the tendon of the biceps femoris to the adjacent soft tissues rather than the proximal tibia. The mean age was significantly higher in patients with varus laxity than in those without laxity and may be related to a greater regeneration and healing potential in younger patients. This difference has not been reported previously. Although the patients were asymptomatic, collateral ligament injury is recognised as a risk factor for knee osteoarthritis [22–24]. Reattaching the fibular collateral ligament and the tendon of the biceps femoris to the proximal tibial metaphysis is likely to prevent or reduce the severity of knee laxity [20, 21], though a comparative study would be required to confirm this conclusively. However, neither the present study nor others have demonstrated anterior translation of the tibia following resection of the fibular head with the exception of that reported by Draganich et al.; this difference is likely to be due to the objective instrumented assessment by that team, whereas the other reports have relied on clinical assessment of the knee alone.

There was no restriction of movement of the ankle and knee joints in our study, in accordance with most other studies [18, 19, 25, 26].

Lee et al. demonstrated definite differences on gait analysis between donor and normal legs in his series of 10 patients of whom four underwent fibula head resection. He attributed this to weakness of deep muscles from loss of their normal origin and a change in load transmission through the fibula [27]. Dieckmann et al. [9] reported a high-stepping gait following resection of the common peroneal nerve for malignant lesions of the proximal fibula. In Draganich et al.’s series of six patients, gait analysis failed to demonstrate significant differences in gait and motion of the knee in comparison with normal controls despite a significant increase in knee ligament laxity [7]. Bickels et al. [8] reported 95 % of normal gait function following Type I (marginal) resection and 77 % of normal gait function following Type II (wide) resection.

None of the patients in our series had gait disturbance on clinical examination. This is likely to be due to the absence of malignant or aggressive lesions and hence the need for extensive dissection in our series. We acknowledge that quantitative gait analysis may demonstrate minor functional losses not perceived by the patient or the examiner [28].

There have been reports of regeneration of the resected fibular shaft [11, 26, 29, 30]; to our knowledge, there have been no reports of regeneration of the proximal third of fibula. Bettin et al. [26] studied regeneration of the donor site in 53 patients undergoing transplantation of the fibular shaft and found an age < 15 years to be the only predictor of regeneration. Herranz et al. have associated absence or incomplete regeneration of the fibula with the development of valgus deformity of the tibia in children and adolescents. They recommend preserving the periosteum to encourage complete fibular regeneration [11]. The scarcity of regeneration of the ends of the fibula can be understood through a detailed knowledge of the blood supply of the fibula and its periosteum. The lower third and majority of the upper third of fibula are subcutaneous, and hence, the periosteum relies more on the nutrient artery than on adjacent muscle arteries for blood supply. When a fibular segment is removed in these areas, the main source of blood supply (nutrient artery) to the periosteum is therefore disrupted and ischaemia leads to failure of regeneration in the defect. In contrast, the middle third of the fibula is richly surrounded by muscle origins and has an abundance of muscle-periosteal anastomoses. With the disruption of the nutrient artery through removal of a fibular segment, the muscle-periosteal anastomoses dilate and regeneration of the fibula is therefore more likely to occur [31].

Hsu et al. and others have also reported on the higher regeneration potential of those of a younger age [26, 31]. The unusual presence of proximal fibula regeneration in a single case in the present study is likely to be due to a favourable balance between interruption of the nutrient artery and the regeneration potential of the vessels and periosteum of a young patient.

Multiple authors have reported proximal migration of the fibula, ankle valgus deformity and tibial diaphyseal valgus deformity after resecting the fibular shaft [11, 31–34]. Little is known if such changes also occur following resection of the fibular head. None of the patients in our series, including children, had any of the above changes. This could be because all patients had more than a 50 % remnant fibula; only 10 % of the fibula has been found to be essential distally to maintain ankle stability [35].

## Conclusion

Donor-site morbidity following simple and marginal resection of the proximal fibula is acceptable. Older patients appear to have a significantly higher risk of demonstrable clinical varus laxity. A longer follow-up would be required to determine whether the asymptomatic instability would lead to early knee arthritis. Proximal fibula resection in children appears to be safe.
